# Insecticide Resistance and Its Management in Two Invasive Cryptic Species of *Bemisia tabaci* in China

**DOI:** 10.3390/ijms24076048

**Published:** 2023-03-23

**Authors:** Qian Wang, Chen Luo, Ran Wang

**Affiliations:** Institute of Plant Protection, Beijing Academy of Agriculture and Forestry Sciences, Beijing 100097, China

**Keywords:** *Bemisia tabaci*, invasive whiteflies, insecticide resistance, pest control, resistance management

## Abstract

The sweet potato whitefly *Bemisia tabaci* is a major agricultural pest with a wide host range throughout the world. The species designation for *B. tabaci* includes numerous distinct cryptic species or biotypes. Two invasive *B. tabaci* biotypes, MEAM1 (B) and MED (Q), were found in China at the end of the 20th century and at the beginning of the 21st century. MEAM1 (B) and MED (Q) show higher pesticide resistance levels than native strains, and the levels of resistance vary with changes in insecticide selection pressure. Recent studies have revealed metabolic resistance mechanisms and target site mutations in invasive *B. tabaci* strains that render them resistant to a range of insecticides and have uncovered the frequency of these resistance-related mutations in *B. tabaci* populations in China. Novel pest control agents, such as RNA-based pesticides and nano-pesticides, have achieved effective control effects in the laboratory and are expected to be applied for field control of *B. tabaci* in the future. In this review, we discuss the mechanisms of resistance developed by these invasive *B. tabaci* populations since their invasion into China. We also provide suggestions for ecologically sound and efficient *B. tabaci* control.

## 1. Introduction: The Whitefly *Bemisia tabaci*

The sweet potato whitefly *B. tabaci* is an insect pest that causes serious agricultural and economic losses throughout the world [1]. This pest damages host plants by feeding on phloem sap and secreting honeydew on leaves and fruits. It serves as a vector for more than 100 plant viruses, including causative agents of serious plant diseases, such as cotton leaf curl virus (CLCV), cassava mosaic disease (CMD), and cassava brown streak disease (CBSD). *B. tabaci* encompasses numerous distinct cryptic species or biotypes; biotypes B (Middle East Asia Minor 1 [MEAM1]) and Q (Mediterranean [MED]) are by far the most widespread [2].

*B. tabaci* has been recorded as causing damage in China since 1949 [3], but increasingly serious damage has occurred since the beginning of the 21st century. A sudden outbreak of *B. tabaci* in China attracted the attention of researchers, and the populations involved were identified in 2002 as the biotype MEAM1 (B). This identification was accomplished by sequencing a fragment of the mitochondrial cytochrome oxidase I gene (mtCOI) [4]. From the time of invasion to the determination of the relevant biotype, MEAM1 (B) rapidly spread to more than ten provinces in China and caused serious damage to a variety of vegetables, flowers, garden plants, and cash crops. MEAM1 (B) showed the ability to outcompete and replace non-B populations over a short period of time.

At the beginning of the 21st century, MED (Q) was first detected in Henan Province and Beijing using the same mtCOI sequencing method [5]. Since then, MED (Q) has been continually reported in different regions of China, rapidly displacing MEAM1 (B) in most locations [6]. Field biotype investigations revealed that the proportion of MED (Q) individuals in China increased tremendously from 2003 to 2014 [7]. Most field-collected populations from different cities and regions in 2011 were identified as MED (Q), but the populations in several areas were of mixed Q and B biotypes [8]. In 2018, MED (Q) was distributed across all areas of Xinjiang [9]. By 2020, MED (Q) was the predominant cryptic species in most areas of China [10].

This process involved two sequential rapid invasions of novel *B. tabaci* biotypes to replace local populations; MEAM1 (B) first displaced non-B populations over a short time period, then MED (Q) rapidly spread to almost all provinces and cities in China and established population dominance [8,11]. The competitive substitution between biotypes was considered to be related to several factors: fitness costs, insecticide resistance, viral vector capacity, and host plant adaptation range [12]. MEAM1 (B) has significant survival advantages compared to MED (Q), but it is often replaced by MED (Q) in the field. MED (Q) shows higher levels of resistance to insecticide than MEAM1 (B), indicating that the insecticide resistance advantage of MED (Q) can compensate for survival disadvantages in the field.

## 2. *B. tabaci* Resistance to Pesticides of Different Classes

**Figure 1:**
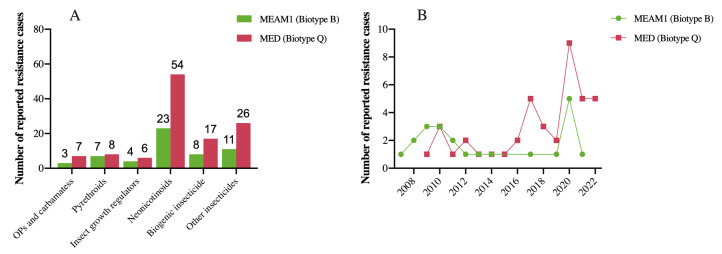
Recorded insecticide resistance cases among invasive *Bemisia tabaci* biotypes in China: (**A**) reported insecticide resistance cases by insecticide category, and (**B**) reported resistance cases in China over time.

The high level of pesticide resistance in invasive *B. tabaci* is considered as one of the important factors driving their rapid spread and replacement of native biotypes. Reported insect resistance mechanisms identified in *B. tabaci* populations in China are shown in Table 1. Single-nucleotide polymorphism (SNP) of cytochrome P450 monooxygenases (P450s) may cause an overexpression of P450-dependent monooxygenases, thus enhancing insecticide resistance [13]. However, the broad trends underlying the development of insecticide resistance and the specific resistance mechanisms used by invasive *B. tabaci* biotypes have remained largely unclear. Clarification of these points would allow the formulation of clear strategies to slow the development and spread of insecticide resistance and improve pesticide efficiency in the field. In Figure 1, we summarize the state of known insecticide resistance in invasive *B. tabaci* biotypes based on published reports from different locations throughout China.

### 2.1. Organophosphates (OPs) and Carbamates

Organophosphorus and carbamate insecticides were released in the 1950s. They are acetylcholinesterase inhibitors that have been applied for many years in China to control multiple pests. *B. tabaci* has developed a range of resistance levels to these pesticides [14]. In general, the development of insecticide resistance is closely associated with the amount and duration of insecticide application. Field MED (Q) populations in Fujian Province displayed high resistance to methamidophos and chlorpyrifos, which had been applied in the field for many years. However, in 2004, there was no apparent resistance of MED (Q) individuals in this area to phoxim, which had only been applied in the area since 2002 [15]. In 2006 and 2007, MEAM1 (B) and MED (Q) individuals in Guangzhou showed significantly higher resistance levels to acephate and methomyl than that of the local Cv population, which was collected in 2007 from the ornamental plant *Codiaeum variegatum* [16]. MED (Q) collected from eastern China in 2010 showed low resistance to dichlorvos and no resistance to carbosulfan, and the susceptible reference strain of B-biotype *B. tabaci* had high resistance; the median lethal concentrations (LC_50_ values) were 336 and 441 mg/L for dichlorvos and carbosulfan, respectively [14]. In comparison, these two pesticides still had high toxicities to field MED (Q) strains. Both MED (Q) and MEAM1 (B) individuals from China displayed extremely low sensitivity to chlorpyrifos [17,18]. However, China banned chlorpyrifos use on vegetables, and this insecticide has therefore not been in use in the field since 2016 [19]. Overall, MEAM1 (B) and MED (Q) have developed high resistance to most OPs and carbamate insecticides, but the resistance levels vary between populations and specific insecticides.

Target site insensitivity and detoxification enzyme activity are the major mechanisms by which *B. tabaci* exert resistance to OPs [14]. Esterase is the primary detoxification enzyme that participates in chlorpyrifos metabolism. A chlorpyrifos-resistant strain showed significantly higher esterase activity than a sensitive strain did, but there were no differences in acetylcholine esterase (AChE) activity [20]. An F392W mutation in *Ace1*, a point mutation in an ace1-type acetylcholinesterase, was shown to be responsible for OP resistance in *B. tabaci* in Israel [21]. However, this mutation was present in both chlorpyrifos-resistant and chlorpyrifos-susceptible strains from Nanjing and in six field-collected populations from elsewhere in China, indicating that carbamate insecticide resistance is widespread [20]. An investigation of another *Ace1* mutation (F331W) that was associated with OP resistance revealed that all analyzed samples were homozygous for the mutation, indicating that the F331W mutation was fixed in wild whitefly populations across China [18].

### 2.2. Pyrethroids

Pyrethroid insecticides were released in the late 1960s. They were gradually but widely adopted due to their higher toxicity and lower required dosages compared to the older generation of organophosphorus insecticides. At the beginning of the MEAM1 (B) invasion into China, this biotype showed high resistance to pyrethroids, with resistance levels up to 1000-fold higher compared to the local populations [22]. Most invasive biotypes showed varying degrees of resistance to pyrethroids, with the exception of cypermethrin, to which MED (Q) populations from eastern China in 2010 were susceptible [14]. Both MEAM1 (B) and MED (Q) showed higher resistance to beta-cypermethrin than the native Cv population did [16]. By 2007–2008, both invasive biotypes from different regions of China were resistant to bifenthrin and cypermethrin, with no differences between the biotypes [23]. By 2019, most field populations in China had developed bifenthrin resistance [10]. Pyrethroid resistance generally functions through target site insensitivity or increased degradation activity of enzymes such as hydrolases and mixed-function oxidases (MFOs) [15]. Two specific point mutations in the gene encoding para-type voltage-gated sodium channel (VGSC), L925I and T929V, are associated with pyrethroid resistance in *B. tabaci* [24].

### 2.3. Insect Growth Regulators (IGRs)

Buprofezin and pyriproxyfen are IGRs that have long been used for *B. tabaci* control. Buprofezin is a chitin biosynthesis inhibitor, whereas pyriproxyfen is an analog of juvenile whitefly hormone. Invasive *B. tabaci* in China has developed a range of resistance levels to pyriproxyfen. MEAM1 (B) sampled from Xinjiang in 2004–2005 showed a pyriproxyfen resistance factor (RF) of 22–37 (LC_50_ = 0.022–0.037 mg/L), although this pesticide had not been in routine use in Chinese agriculture at that time [22]. Pyriproxyfen had an LC_50_ value of 1461 mg/L in lab-raised MEAM1 (B) adults descended from individuals collected in Beijing in 2000; the LC_50_ value was 8832 mg/L for lab-raised MED (Q) adults descended from individuals collected in Beijing in 2009, demonstrating a significant increase in resistance [25]. Furthermore, adults were significantly more pyriproxyfen-resistant than eggs and larvae in both populations, whereas eggs and adults showed significantly higher buprofezin resistance than larvae [25]. MED (Q) larvae collected from multiple Chinese provinces in 2013–2014 showed low to moderate levels of pyriproxyfen resistance, but the resistance levels markedly increased in 2014 [7]. From 2015 to 2018, MEAM1 (B) and MED (Q) individuals collected from regions throughout China showed low to high pyriproxyfen resistance, and resistance was dependent on developmental stage [19,26].

### 2.4. Neonicotinoids

**Figure 2:**
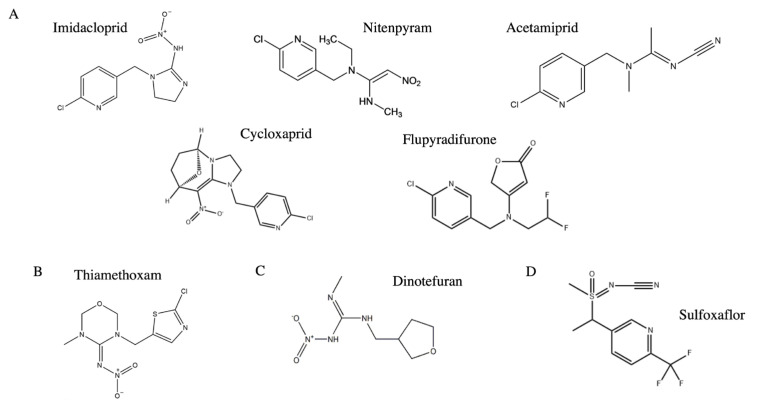
Chemical structure of neonicotinoid insecticides applied in China: (**A**) chloronicotinyls; (**B**) thianicotinyls; (**C**) nitroguanidines; and (**D**) sulfoximines.

Neonicotinoids have played a key role in *B. tabaci* control in the field for many years. This class of pesticides can be divided into four categories based on their chemical structure: chloronicotinyls, thianicotinyls, nitroguanidines, and sulfoximines (Figure 2). Neonicotinoids are nicotinic acetylcholine receptor (nAChR) competitive modulators, of which four generations have been released over time. At the beginning of the MEAM1 (B) invasion, this biotype showed moderate resistance to the first and second generations of neonicotinoid pesticides. The LC_50_ values for imidacloprid were five- to seven-fold higher in MEAM1 (B) and MED (Q) than in the local Cv populations [16]. In a lab-reared population of MEAM1 (B), thiamethoxam resistance developed slowly prior to the 17th generation and then rapidly increased. Thiamethoxam resistance in *B. tabaci* has high fitness costs, as demonstrated by the lower intrinsic rate of increase, gross reproductive rate, and net reproductive rate [27]. Field MED (Q) populations have consistently shown much higher resistance levels to imidacloprid, thiamethoxam, and acetamiprid than MEAM1 (B) populations [28]. In 2019, most MED (Q) populations in Xinjiang were susceptible to thiamethoxam, but were moderate to high resistant to imidacloprid [9]. MED (Q) from eastern China showed low to high resistance to the first- and second-generation neonicotinoids, but no resistance to third-generation neonicotinoids from 2013 to 2014 [29]. Flupyradifurone is a butenolide insecticide that has only been applied for a short time in China, but some field populations have already shown low to moderate levels of resistance; however, this resistance is associated with significant fitness costs [30].

Detoxification enzyme activity is a major mechanism of resistance to neonicotinoids. P450 monooxygenase plays a key role in the regulation of resistance; it shows significantly higher activity in neonicotinoid-resistant strains and is overexpressed in response to neonicotinoid stimulation [31,32]. Of the numerous P450 genes, several have been confirmed as being involved in the regulation of neonicotinoid resistance. These include cytochrome P450 (CYP) genes, such as CYP6CM1, CYP4C64, and CYP6CX4, and the glutathione-*S*-transferase (GST) s2 and d7 proteins (GSTs2 and GSTd7) [8,28,33,34,35,36]. CYP6CM1 expression levels are closely related to the resistance factors of *B. tabaci* populations to imidacloprid, thiamethoxam, acetamiprid, and pymetrozine [8,28]. CYP6CM1 is most closely associated with the regulation of imidacloprid resistance. A simulated binding model of CYP6CM1vQ with imidacloprid shows a relatively stable interaction, with two stable N-H···N H-bonds and a strong cation–π interaction between Arg225 and imidacloprid; in contrast, the interaction of CYP6CM1vQ with dinotefuran is weak [33]. Reducing CYP6CM1 mRNA levels is sufficient to decrease the resistance of an imidacloprid-tolerant strain to imidacloprid. The basic leucine zipper (bZIP) transcription factor, cAMP-response element-binding protein (CREB), directly regulates CYP6CM1 expression, and the extracellular signal-related kinase (ERK) and p38 mitogen-activated protein kinase (MAPK) signaling pathways activate CREB transcription [35]. The relative expression levels of CYP6CX4 and GSTs2 in a flupyradifurone-tolerant *B. tabaci* strain were markedly higher than those in a susceptible strain, and decreases in CYP6CX4 and GSTs2 expression resulted in a significant increase in mortality rate in response to flupyradifurone treatment [34]. CYP4C64 was strongly overexpressed in thiamethoxam-resistant strains, and knocking down CYP4C64 significantly reduced *B. tabaci* resistance to thiamethoxam. A T206A mutation in the 5′ untranslated region (UTR) enhanced CYP4C64 expression in thiamethoxam-resistant strains [37]. The expression levels of several other P450 genes are correlated with neonicotinoid treatment; for example, CYP4CS3, CYP6CX5, and CYP6DW2 were significantly up-regulated in imidacloprid- and acetamiprid-tolerant *B. tabaci* strains [32]. Activities of carboxylesterase (CarE) and GST were also reportedly related to thiamethoxam resistance [38,39], indicating that *B. tabaci* resistance to neonicotinoid insecticides may be a combined result of the activities of multiple detoxification enzymes rather than a single enzyme.

### 2.5. Biogenic Insecticide

Avermectins are a class of antibiotic insecticides produced as specialized metabolites by *Streptomyces*; the avermectin compound abamectin is widely used across China and remains an effective pesticide for *B. tabaci* control in most regions [19]. For many years, abamectin displayed high toxicity to *B. tabaci* MEAM1 (B) and MED (Q). Continuous selection in the laboratory revealed that the development of abamectin resistance in *B. tabaci* was relatively slow [40]. Until 2018, nearly all tested field strains displayed no resistance to abamectin [12,14,23,25]. However, in 2016–2017, MEAM1 (B) and MED (Q) collected from Shandong Province displayed moderate resistance to abamectin, suggesting that some populations may have an increased chance of developing resistance [19]. Furthermore, the eggs and larvae of laboratory strains showed higher resistance to abamectin than adults [25]. A new semi-synthetic antibiotic insecticide, emamectin benzoate, is highly effective, with increased efficiency and decreased toxicity compared to avermectin. Field strains from Fujian Province showed low resistance to emamectin benzoate [15]. Field strains from multiple regions in China showed minimal or no resistance to another new biogenic insecticide, spinosad, or its derivative, spinetoram [12,15,41]. In general, biological insecticides show promise as efficient insecticides for the control of field populations of *B. tabaci* in China. However, although abamectin resistance development is slow, the risk of increased resistance should still be addressed. Another new biogenic insecticide, afidopyropen, is a semi-synthetic derivative of pyripyropene A, a fermentation product of *Aspergillus fumigatus* Fresenius (Eurotiales: Trichocomaceae). Because it is an insecticide that has been used for a relatively short time in China, afidopyropen has been proven to have high toxicity against *B. tabaci* MEAM1 (B) and MED (Q) in all field populations tested [42]. Resistance selection experiments showed that resistance to afidopyropen in one strain was stably increased from the second to the sixth generation. Additionally, this resistance was subsequently stabilized between the seventh and tenth generations [43]. Thus, in contrast to abamectin, *B. tabaci* is expected to rapidly develop resistance to afidopyropen when exposed to this insecticide within several generations.

The enhanced metabolic detoxification systems found in insects may be the main reason for the observed resistance of *B. tabaci* to these insecticides. In an abamectin-resistant MEAM1 (B) strain, the oxidase inhibitor piperonyl butoxide (PBO) and the GST inhibitor diethyl maleate (DEM) produce significant synergistic effects on abamectin, indicating that enhanced metabolism mediated by P450 monooxygenase and GST activity, but not esterase activity, may be involved in abamectin resistance [40]. In an afidopyropen-resistant strain, PBO also showed significant synergism with afidopyropen, suggesting that oxidative metabolism may be one mechanism that results in afidopyropen resistance [43].

### 2.6. Other Insecticides

Cyantraniliprole is a second-generation anthranilic diamide product that provides cross-spectrum control of chewing and sucking insect pests. From 2012 to 2016, field populations collected from several regions in China showed little to no cyantraniliprole resistance [7,25]. However, since 2018, populations in some regions have begun to develop high resistance [10,18,44]. In one cyantraniliprole-resistant strain, P450 activity was markedly higher than that in susceptible strains, although esterase and GST activities were not significantly different between the two strains [44]. Fipronil is a phenylpyrazole insecticide, to which field populations in China have shown low to moderate resistance [12,15]. The major inhibitory neurotransmitter in insects is γ-aminobutyric acid (GABA); fipronil blocks a GABA-activated chloride channel, causing hyperexcitation and convulsions. Pymetrozine is a pyridine azomethine derivative, and resistance to this insecticide varies from low to high depending on the field populations sampled [11,18,19,28]. In the laboratory, pymetrozine resistance slowly increased from the first to the tenth generation, then rapidly increased from the eleventh to the eighteenth generation [45]. Long-term application of pymetrozine would, therefore, be expected to lead to rapid development of resistance in both MEAM1 (B) and MED (Q) populations. CYP6CM1 expression is closely related to pymetrozine resistance factors across *B. tabaci* populations [28].

### 2.7. Cross-Resistance

As mentioned above, insecticide resistance occurs via two distinct mechanisms: target-site point mutations and increased detoxification effects [33]. Insecticides that share target sites are more likely to form cross-resistance, but increased metabolic resistance may cause cross-resistance between different types of pesticides. An abamectin-tolerant *B. tabaci* strain showed obvious cross-resistance to the abamectin analogue, emamectin benzoate, and to imidacloprid, but not to fipronil [40]. Elevated metabolic detoxification is likely responsible for this cross-resistance because abamectin does not share target sites with imidacloprid. Cross-resistance to neonicotinoid insecticides is common. A thiamethoxam-resistant strain showed varying levels of cross-resistance to imidacloprid, acetamiprid, and nitenpyram, but not to abamectin or bifenthrin [38,39]. An imidacloprid-resistant strain showed high levels of cross-resistance to neonicotinoids, but not to cypermethrin or abamectin [31]. However, some neonicotinoid-resistant strains have displayed varying degrees of cross-resistance to other pesticides (such as abamectin and carbosulfan), which may be caused by elevated metabolic detoxification [38]. A flupyradifurone-resistant strain showed high cross-resistance to imidacloprid but no cross-resistance to thiamethoxam, acetamiprid, nitenpyram, or sulfoxaflor [34]. This suggests that the regulatory mechanisms used to resist damage caused by neonicotinoid insecticides are slightly different. A cyantraniliprole-resistant strain had no cross-resistance to imidacloprid, thiamethoxam, abamectin, sulfoxaflor, or bifenthrin [46]. A pymetrozine-resistant strain displayed low levels of cross-resistance to acetamiprid, imidacloprid, nitenpyram, and thiamethoxam, but no cross-resistance to abamectin or chlorpyrifos [45]. There was no cross-resistance between afidopyropen and pymetrozine in *B. tabaci*, even though these pesticides target the transient receptor potential vanilloid (TRPV) channel of insects, suggesting that afidopyropen may more effectively target the TRPV channel [42]. Furthermore, afidopyropen-resistant MED (Q) individuals showed significant cross-resistance to sulfoxaflor, but little cross-resistance to cyantraniliprole, flupyradifurone, imidacloprid, or thiamethoxam [43].

**Table 1:** Reported insecticide resistance mechanisms of invasive *Bemisia tabaci* biotypes in China.

Insecticide	Resistance Mechanism	Associated Gene	References
**Organophosphates (ops)** Methamidophos Phoxim Chlorpyrifos Profenofos Acephate Malathion	Target resistance	Acetylcholinesterase ace1 gene	[14,18,20,21]
**Pyrethroids** Bifenthrin Cypermethrin	Target resistance	Para-type voltage-gated sodium channel gene	[14,18]
**Neonicotinoids** Dinotefuran Imidacloprid Thiamethoxam Acetamiprid Sulfoxaflor Flupyradifurone Acetamiprid	Metabolic resistance	Cytochrome P450 gene; glutathione-S-transferase gene (GST); nicotinic acetylcholine receptor β1 subunit gene (Btβ1); and ATP-binding cassette subfamily G member 3 gene (ABCG3)	[8,9,18,28,29,32,33,34,35,39,47]
**Biogenic insecticides** Abamectin Afidopyropen	Metabolic resistance	Cytochrome P450 gene and glutathione-S-transferase gene (GST)	[40,48]
**Other insecticides** Pymetrozine Cyantraniliprole	Metabolic resistance	Cytochrome P450 gene	[28]

## 3. Management of *B. tabaci* Resistance

### 3.1. Insecticide Rotation

Rotating insecticides is the most effective method to delay the evolution of insecticide resistance and to regain pest susceptibility as part of an insecticide resistance management (IRM) program. Under pesticide selection pressure, *B. tabaci* resistance increases at different rates, depending on the pesticide. In the absence of a pesticide, resistance declines rapidly [30,43]. Selection of the most appropriate chemical insecticide for a given situation should take into consideration the available officially registered products and various parameters, such as crop stage, post-harvest interval, presence of beneficial insects or pollinators, pest infestation level, presence of other coexisting pests, and pesticide resistance history. The *B. tabaci* generation time ranges from 24 to 30 d; insecticides with different modes of action must, therefore, be rotated for field application every 30 d. All applications should strictly follow the relevant product label with regard to the rate, target pest, and the application method and frequency.

### 3.2. Improvement in Insecticide Efficiency

The development of insecticide resistance in pests is closely related to long-term and large-scale pesticide application [1,11]. The duration of use of a single insecticide can be interrupted by insecticide rotation, thus slowing the rate of resistance development [25,26]. Reducing the dosage of pesticide application can also decrease the development of resistance; to this end, some methods can be used to improve pesticide efficiency, such as the addition of synergists. The insecticide mixture approach has been thoroughly investigated in numerous pest systems, but it remains controversial. Therefore, the efficacy of mixing pesticides should continue to be assessed based on relevant research.

### 3.3. Synergists

Enzyme inhibitors may improve pesticide sensitivity in *B. tabaci* field populations to varying degrees [15]. Three common inhibitors have significant synergistic effects on pesticides: PBO, DEM, and the esterase inhibitor triphenyl phosphate (TPP). AChE activity is strongly inhibited by PBO, but not by TPP or DEM. In both laboratory and field strains of *B. tabaci*, GST activity is significantly inhibited in vivo by DEM [38]. Rational use of synergists can effectively improve the efficiency of some metabolizable insecticides; the effects of various synergists are closely related to pesticide metabolic mechanisms. PBO can play a synergistic role with numerous pesticides, such as thiamethoxam, imidacloprid, acetamiprid, cyantraniliprole, flupyradifurone, and afidopyropen [31,32,34,38,43,44]. This indicates that oxidases have a key role in the metabolism of many pesticides. Furthermore, TPP has significant synergism with thiamethoxam and chlorpyrifos, confirming that enhanced esterase activity is at least partially responsible for resistance to these pesticides [20,38].

### 3.4. Bacteriostatic and Insecticidal Compounds

Symbionts are ubiquitous in insects. Some participate in the synthesis of essential amino acids, forming a stable symbiotic relationship with the host [49]. The symbionts of various insects are known to improve host insecticide resistance through direct degradation or indirect regulatory mechanisms [50,51,52,53]. *B. tabaci* harbors the primary (P-) endosymbiont, *Candidatus* Portiera aleyrodidarum (Oceanospirillales), in bacteriocytes. Seven S-endosymbionts have also been identified to date, namely *Hamiltonella* (Enterobacteriaceae), *Arsenophonus* (Enterobacteriaceae), *Wolbachia* (Rickettsiales), *Rickettsia* (Rickettsiales), *Cardinium* (Bacteroidetes), *Fritschea* (Chlamydiales), and *Hemipteriphilus* (Proteobacteria) [54]. Several endosymbionts are reported to be associated with insecticide resistance in *B. tabaci*; however, the relevance of endosymbionts to pesticide resistance appears to vary between *B. tabaci* populations and to be dependent on the insecticide used [55,56]. Although the mechanism by which *B. tabaci* endosymbionts participate in the regulation of host pesticide resistance remains unclear, studies of similar systems have revealed relevant mechanisms in *Riptortus pedestris* [50], *Bactrocera dorsalis* [51], and *Nilaparvata lugens* [52]. Future research may uncover effective measures of decreasing *B. tabaci* insecticide resistance by regulating symbiotic bacteria, thereby providing novel strategies for managing *B. tabaci* resistance.

### 3.5. New Prevention and Control Technology

In recent years, pest control strategies based on RNA interference (RNAi) have been rapidly developed. RNA molecules used to control pests are called RNA-based pesticides; such RNAi agents can be used to inhibit or control agricultural pests by silencing the expression of important pest genes. RNA-based pesticides are single- or double-stranded polynucleotide segments that bind specific mRNAs corresponding to the target genes within a pest, causing transcript degradation or translation inhibition [57]. This interferes with normal growth of the target organism to minimize damage to the host plant. Compared with traditional small-molecule pesticides, RNA-based pesticides have several distinct advantages, including a strong target specificity, a lack of toxic residues (i.e., relative environmental safety), and a low development cost [58]. This method has been tested for *B. tabaci* control; RNAi of BtTPS1 and BtTPS2 significantly increased mortality and influenced the expression of target genes involved in energy metabolism and chitin biosynthesis in adults [59]. Nuclear receptors (NRs) play essential roles in diverse biological processes, such as insect metamorphosis. RNAi of some NRs causes malformation phenotypes in *B. tabaci* MED (Q), and several other NRs are potential targets for this pest control method due to their important roles in insect development [60]. Additionally, RNAi of genes related to pesticide resistance could promote pesticide sensitivity, thereby improving insecticide efficiency. For example, RNAi of CYP6CM1 is sufficient to increase imidacloprid sensitivity in a resistant strain [35]. Furthermore, RNAi of Inactive (*Iav*) together with Nanchung (*Nan*), a component of TRPV channel, increases MED (Q) sensitivity to afidopyropen [61]. Although RNAi technology is in development for commercial pest control, obstacles to its large-scale application remain. These include low efficiency of RNA delivery to target cells, low efficiency of target gene silencing, dose-limiting toxicity, insufficient interference efficiency, and low RNA molecule stability [62]. Despite these challenges, RNAi has tremendous potential for application in agricultural pest control.

At present, most pesticides in China are emulsifiable concentrates and wettable powders that have poor water dispersion, low biological activity, and low effective utilization [63]. The poor water solubility of most effective pesticide components is one of the most important factors inhibiting the improvement of effective pesticide utilization. Nano-pesticides are small in size but have large surface areas, and they can therefore improve water dispersibility and expand the contact area between pesticides and target pests, thereby increasing pesticide bioavailability. However, the small size, surface modifiability, and other desirable characteristics of nano-pesticides can also promote absorption and transport of these pesticides by plants; specifically, hydrophobic nano-pesticides may be absorbed during water uptake [64]. Leaf surfaces generally exhibit some hydrophobicity, which makes it difficult for pesticides to adhere; they tend to slide or wash off, minimizing pesticide application efficiency. Adding specific groups or changing the charge properties of nano-pesticides that have surface modifiability can enhance leaf adhesion [64]. Compared to traditional pesticides, nano-pesticides are, therefore, easier to keep in contact with plant leaves and stems, which prolongs the effective period and improves utilization. Furthermore, nanocarriers can effectively improve the environmental stability of active ingredients. They can also be used to build a controlled release system that responds to external pH, redox reactions, enzyme activity, light, temperature, or other factors, which can reduce the required dosage and frequency of pesticide application and increase the utilization rate. Although the safety of nano-pesticides for the environment and non-target organisms is unclear, they still represent a promising research area for pest control.

## 4. Conclusions

Two invasive *B. tabaci* biotypes, MEAM1 (B) and MED (Q), have rapidly spread in China. The speed of their spread is considered to be closely related to fitness costs, insecticide resistance, viral vector capacity, and host plant adaptation range. Chemical insecticides have served as the main method for controlling *B. tabaci* populations for many years, and they will likely remain so in the near future. Controlling the development and spread of pesticide resistance and improving insecticide efficiency will, therefore, be critical aspects of *B. tabaci* control in the future. MEAM1 (B) and MED (Q) populations had varying degrees of resistance to numerous traditional insecticides at the onset of their invasion of China. They have gradually developed additional resistance to new pesticides as such compounds are made available and applied in the field. Research into the resistance mechanisms used by *B. tabaci* in China has revealed several mutation sites related to OP and pyrethroid resistance. These mutation sites are now widely found throughout *B. tabaci* populations in China. Increases in detoxification enzyme activity have been established as key factors underlying most insecticide resistance, such as resistance to neonicotinoids. Insecticide rotation is a critical strategy to manage pesticide resistance. The continuous application of similar insecticides inevitably leads to rapid increases in insecticide resistance; conversely, reducing insecticide selection pressure will rapidly decrease resistance development. Rational use of synergists can also effectively improve pesticide efficiency and reduce the dosage required. Other pest control strategies, such as inhibiting resistance-promoting endosymbionts or using novel pesticides such as RNAi or nano-pesticides, are promising approaches currently in development for effective pest control.

## Data Availability

All relevant data are available from the corresponding author on request (wangran@ipepbaafs.cn).

## References

[1] Horowitz A.R., Ghanim M., Roditakis E., Nauen R., Ishaaya I. (2020). Insecticide resistance and its management in *Bemisia tabaci* species. J. Pest Sci..

[2] Wang X., Li P., Liu S. (2017). Whitefly interactions with plants. Curr. Opin. Insect Sci..

[3] Zhou Y. (1949). Directory of whiteflies in China. Chin. J. Entomol..

[4] Luo C., Yao Y., Wang R., Yan F., Hu D., Zhang Z. (2002). The use of mitochondrial cytochrome oxidaseI (mtCOI) gene sequences for the identification of biotypes of *Bemisia tabaci* (Gennadius)in China. Acta Entomol. Sin..

[5] Chu D., Zhang Y.-J., Brown J.K., Cong B., Xu B.-Y., Wu Q.-J., Zhu G.-R. (2006). The Introduction Of The Exotic Q Biotype Of *Bemisia Tabaci* From The Mediterranean Region Into China On Ornamental Crops. Fla. Entomol..

[6] Pan H., Chu D., Ge D., Wang S., Wu Q., Xie W., Jiao X., Liu B., Yang X., Yang N. (2011). Further spread of and domination by *Bemisia tabaci* (Hemiptera: Aleyrodidae) biotype Q on field crops in China. J. Econ. Entomol..

[7] Zheng H., Xie W., Wang S., Wu Q., Zhou X., Zhang Y. (2017). Dynamic monitoring (B versus Q) and further resistance status of Q-type *Bemisia tabaci* in China. Crop Prot..

[8] Yang X., Xie W., Wang S.L., Wu Q.J., Pan H.P., Li R.M., Yang N.N., Liu B.M., Xu B.Y., Zhou X. (2013). Two cytochrome P450 genes are involved in imidacloprid resistance in field populations of the whitefly, *Bemisia tabaci*, in China. Pestic. Biochem. Physiol..

[9] Wang Q., Wang M., Jia Z., Ahmat T., Xie L., Jiang W. (2020). Resistance to neonicotinoid insecticides and expression changes of eighteen cytochrome P450 genes in field populations of *Bemisia tabaci* from Xinjiang, China. Entomol. Res..

[10] Guo L., Lv H., Tan D., Liang N., Guo C., Chu D. (2020). Resistance to insecticides in the field and baseline susceptibility to cyclaniliprole of whitefly *Bemisia tabaci* (Gennadius) in China. Crop Prot..

[11] Yao F., Zheng Y., Huang X., Ding X., Zhao J., Desneux N., He Y., Weng Q. (2017). Dynamics of *Bemisia tabaci* biotypes and insecticide resistance in Fujian province in China during 2005–2014. Sci. Rep..

[12] Wang Z., Yan H., Yang Y., Wu Y. (2010). Biotype and insecticide resistance status of the whitefly *Bemisia tabaci* from China. Pest Manag. Sci..

[13] Karunker I., Benting J., Lueke B., Ponge T., Nauen R., Roditakis E., Vontas J., Gorman K., Denholm I., Morin S. (2008). Over-expression of cytochrome P450 CYP6CM1 is associated with high resistance to imidacloprid in the B and Q biotypes of *Bemisia tabaci* (Hemiptera: Aleyrodidae). Insect Biochem. Mol. Biol..

[14] Yuan L., Wang S., Zhou J., Du Y., Zhang Y., Wang J. (2012). Status of insecticide resistance and associated mutations in Q-biotype of whitefly, *Bemisia tabaci*, from eastern China. Crop Prot..

[15] Kang C.Y., Wu G., Miyata T. (2006). Synergism of enzyme inhibitors and mechanisms of insecticide resistance in *Bemisia tabaci* (Gennadius) (Hom., Aleyrodidae). J. Appl. Entomol..

[16] Qiu B., Liu L., Li X., Mathur V., Qin Z., Ren S. (2009). Genetic mutations associated with chemical resistance in the cytochrome P450 genes of invasive and native *Bemisia tabaci* (Hemiptera: Aleyrodidae) populations in China. Insect Sci..

[17] He Y., Zhao J., Zheng Y., Weng Q., Biondi A., Desneux N., Wu K. (2013). Assessment of potential sublethal effects of various insecticides on key biological traits of the tobacco whitefly, *Bemisia tabaci*. Int. J. Biol. Sci..

[18] Wang R., Che W., Wang J., Luo C. (2020). Monitoring insecticide resistance and diagnostics of resistance mechanisms in *Bemisia tabaci* Mediterranean (Q biotype) in China. Pestic. Biochem. Physiol..

[19] Wang F., Liu J., Chen P., Li H., Ma J., Liu Y., Wang K. (2019). *Bemisia tabaci* (Hemiptera: Aleyrodidae) Insecticide Resistance in Shandong Province, China. J. Econ. Entomol..

[20] Zhang N., Liu C., Yang F., Dong S., Han Z. (2011). Resistance mechanisms to chlorpyrifos and F392W mutation frequencies in the acetylcholine esterase ace1 allele of field populations of the tobacco whitefly, *Bemisia tabaci* in China. J. Insect Sci..

[21] Alon M., Alon F., Nauen R., Morin S. (2008). Organophosphates’ resistance in the B-biotype of *Bemisia tabaci* (Hemiptera: Aleyrodidae) is associated with a point mutation in an ace1-type acetylcholinesterase and overexpression of carboxylesterase. Insect Biochem. Mol. Biol..

[22] Ma D., Gorman K., Devine G., Luo W., Denholm I. (2007). The biotype and insecticide-resistance status of whiteflies, *Bemisia tabaci* (Hemiptera: Aleyrodidae), invading cropping systems in Xinjiang Uygur Autonomous Region, northwestern China. Crop Prot..

[23] Luo C., Jones C.M., Devine G., Zhang F., Denholm I., Gorman K. (2010). Insecticide resistance in *Bemisia tabaci* biotype Q (Hemiptera: Aleyrodidae) from China. Crop Prot..

[24] Wei Y., Guan F., Wang R., Qu C., Luo C. (2021). Amplicon sequencing detects mutations associated with pyrethroid resistance in *Bemisia tabaci* (Hemiptera: Aleyrodidae). Pest Manag. Sci..

[25] Xie W., Liu Y., Wang S., Wu Q., Pan H., Yang X., Guo L., Zhang Y. (2014). Sensitivity of *Bemisia tabaci* (Hemiptera: Aleyrodidae) to several new insecticides in China: Effects of insecticide type and whitefly species, strain, and stage. J. Insect Sci..

[26] Zheng H., Xie W., Fu B., Xiao S., Tan X., Ji Y., Cheng J., Wang R., Liu B., Yang X. (2021). Annual analysis of field-evolved insecticide resistance in *Bemisia tabaci* across China. Pest Manag. Sci..

[27] Feng Y., Wu Q., Xu B., Wang S., Chang X., Xie W., Zhang Y. (2009). Fitness costs and morphological change of laboratory-selected thiamethoxam resistance in the B-type *Bemisia tabaci* (Hemiptera: Aleyrodidae). J. Appl. Entomol..

[28] Rao Q., Xu Y.-h., Luo C., Zhang H.-y., Jones C.M., Devine G.J., Gorman K., Denholm I. (2012). Characterisation of Neonicotinoid and Pymetrozine Resistance in Strains of *Bemisia tabaci* (Hemiptera: Aleyrodidae) from China. J. Integr. Agric..

[29] Wang W., Wang S., Han G., Du Y., Wang J. (2017). Lack of cross-resistance between neonicotinoids and sulfoxaflor in field strains of Q-biotype of whitefly, *Bemisia tabaci*, from eastern China. Pestic. Biochem. Physiol..

[30] Wang R., Zhang J., Che W., Wang J., Luo C. (2022). Genetics and fitness costs of resistance to flupyradifurone in *Bemisia tabaci* from China. J. Integr. Agric..

[31] Wang Z., Yao M., Wu Y. (2009). Cross-resistance, inheritance and biochemical mechanisms of imidacloprid resistance in B-biotype *Bemisia tabaci*. Pest Manag. Sci. Former. Pestic. Sci..

[32] Zhou C., Cao Q., Li G., Ma D. (2020). Role of several cytochrome P450s in the resistance and cross-resistance against imidacloprid and acetamiprid of *Bemisia tabaci* (Hemiptera: Aleyrodidae) MEAM1 cryptic species in Xinjiang, China. Pestic. Biochem. Physiol..

[33] Meng X., Zhu C., Feng Y., Li W., Shao X., Xu Z., Cheng J., Li Z. (2016). Computational Insights into the Different Resistance Mechanism of Imidacloprid versus Dinotefuran in *Bemisia tabaci*. J. Agric. Food Chem..

[34] Wang R., Wang J., Zhang J., Che W., Feng H., Luo C. (2020). Characterization of flupyradifurone resistance in the whitefly *Bemisia tabaci* Mediterranean (Q biotype). Pest Manag. Sci..

[35] Yang X., Deng S., Wei X., Yang J., Zhao Q., Yin C., Du T., Guo Z., Xia J., Yang Z. (2020). MAPK-directed activation of the whitefly transcription factor CREB leads to P450-mediated imidacloprid resistance. Proc. Natl. Acad. Sci. USA.

[36] He C., Xie W., Yang X., Wang S., Wu Q., Zhang Y. (2018). Identification of glutathione S-transferases in *Bemisia tabaci* (Hemiptera: Aleyrodidae) and evidence that GSTd7 helps explain the difference in insecticide susceptibility between *B. tabaci* Middle East-Minor Asia 1 and Mediterranean. Insect Mol. Biol..

[37] Yang X., Wei X., Yang J., Du T., Yin C., Fu B., Huang M., Liang J., Gong P., Liu S. (2021). Epitranscriptomic regulation of insecticide resistance. Sci. Adv..

[38] Feng Y., Wu Q., Wang S., Chang X., Xie W., Xu B., Zhang Y. (2010). Cross-resistance study and biochemical mechanisms of thiamethoxam resistance in B-biotype *Bemisia tabaci* (Hemiptera: Aleyrodidae). Pest Manag. Sci. Former. Pestic. Sci..

[39] Yang X., He C., Xie W., Liu Y., Xia J., Yang Z., Guo L., Wen Y., Wang S., Wu Q. (2016). Glutathione S-transferases are involved in thiamethoxam resistance in the field whitefly *Bemisia tabaci* Q (Hemiptera: Aleyrodidae). Pestic. Biochem. Physiol..

[40] Wang L., Wu Y. (2007). Cross-resistance and biochemical mechanisms of abamectin resistance in the B-type *Bemisia tabaci*. J. Appl. Entomol..

[41] Wang S., Zhang Y., Yang X., Xie W., Wu Q. (2017). Resistance Monitoring for Eight Insecticides on the Sweetpotato Whitefly (Hemiptera: Aleyrodidae) in China. J. Econ. Entomol..

[42] Zhang Z., Shi H., Xu W., Liu J., Geng Z., Chu D., Guo L. (2021). Pymetrozine-resistant whitefly *Bemisia tabaci* (Gennadius) populations in China remain susceptible to afidopyropen. Crop Prot..

[43] Wang R., Zhang Q., Zhou X., Zhang M., Yang Q., Su Q., Luo C. (2022). Characterization of Field-Evolved Resistance to Afidopyropen, a Novel Insecticidal Toxin Developed from Microbial Secondary Metabolites, in *Bemisia tabaci*. Toxins.

[44] Wang R., Che W., Wang J., Qu C., Luo C. (2020). Cross-resistance and biochemical mechanism of resistance to cyantraniliprole in a near-isogenic line of whitefly *Bemisia tabaci* Mediterranean (Q biotype). Pestic. Biochem. Physiol..

[45] Wang F., Liu J., Shuai S., Miao C., Chi B., Chen P., Wang K., Li H., Liu Y. (2021). Resistance of *Bemisia tabaci* Mediterranean (Q-biotype) to pymetrozine: Resistance risk assessment, cross-resistance to six other insecticides and detoxification enzyme assay. Pest Manag. Sci..

[46] Wang R., Wang J., Che W., Sun Y., Li W., Luo C. (2019). Characterization of field-evolved resistance to cyantraniliprole in *Bemisia tabaci* MED from China. J. Integr. Agric..

[47] Wang R., Wang J., Che W., Fang Y., Luo C. (2020). Baseline susceptibility and biochemical mechanism of resistance to flupyradifurone in *Bemisia tabaci*. Crop Protect..

[48] Wang R., Fang Y., Che W., Zhang Q., Wang J., Luo C. (2022). Metabolic Resistance in Abamectin-Resistant *Bemisia tabaci* Mediterranean from Northern China. Toxins.

[49] Xie W., Meng Q.S., Wu Q.J., Wang S.L., Yang X., Yang N.N., Li R.M., Jiao X.G., Pan H.P., Liu B.M. (2012). Pyrosequencing the *Bemisia tabaci* transcriptome reveals a highly diverse bacterial community and a robust system for insecticide resistance. PLoS ONE.

[50] Kikuchi Y., Hayatsu M., Hosokawa T., Nagayama A., Tago K., Fukatsu T. (2012). Symbiont-mediated insecticide resistance. Proc. Natl. Acad. Sci. USA.

[51] Cheng D., Guo Z., Riegler M., Xi Z., Liang G., Xu Y. (2017). Gut symbiont enhances insecticide resistance in a significant pest, the oriental fruit fly *Bactrocera dorsalis* (Hendel). Microbiome.

[52] Zhang Y., Cai T., Ren Z., Liu Y., Yuan M., Cai Y., Yu C., Shu R., He S., Li J. (2021). Decline in symbiont-dependent host detoxification metabolism contributes to increased insecticide susceptibility of insects under high temperature. ISME J..

[53] Tang T., Zhang Y., Cai T., Deng X., Liu C., Li J., He S., Li J., Wan H. (2020). Antibiotics increased host insecticide susceptibility via collapsed bacterial symbionts reducing detoxification metabolism in the brown planthopper, *Nilaparvata lugens*. J. Pest Sci..

[54] Brumin M., Lebedev G., Kontsedalov S., Ghanim M. (2020). Levels of the endosymbiont *Rickettsia* in the whitefly *Bemisia tabaci* are influenced by the expression of vitellogenin. Insect Mol. Biol..

[55] Kontsedalov S., Zchori-Fein E., Chiel E., Gottlieb Y., Inbar M., Ghanim M. (2008). The presence of *Rickettsia* is associated with increased susceptibility of *Bemisia tabaci* (Homoptera: Aleyrodidae) to insecticides. Pest Manag. Sci. Former. Pestic. Sci..

[56] Cao T., Yuan M., Yang K., Guo L., Chu D. (2021). Influences of endosymbiont *Cardinium* on the insecticide tolerance of *Bemisia tabaci* MED (Hemiptera: Aleyrodidae). Acta Entomol. Sin..

[57] Zhang J., Khan S.A., Heckel D.G., Bock R. (2017). Next-Generation Insect-Resistant Plants: RNAi-Mediated Crop Protection. Trends Biotechnol..

[58] Wang Z., Gao X., Ma D., Zhong S., Liu X., Xin Z. (2019). Nucleic acid pesticides—The new plant protection products with great potential. Chin. J. Pestic. Sci..

[59] Gong C., Yang Z., Hu Y., Wu Q., Wang S., Guo Z., Zhang Y. (2022). Silencing of the BtTPS genes by transgenic plant-mediated RNAi to control *Bemisia tabaci* MED. Pest Manag. Sci.

[60] He C., Liu S., Liang J., Zeng Y., Wang S., Wu Q., Xie W., Zhang Y. (2020). Genome-wide identification and analysis of nuclear receptors genes for lethal screening against *Bemisia tabaci* Q. Pest Manag. Sci.

[61] Wang R., Gao B., Zhang Q., Qu C., Luo C. (2022). Knockdown of TRPV gene Nanchung decreases resistance to the novel pyropene insecticide, afidopyropen, in *Bemisia tabaci*. Int. J. Biol. Macromol..

[62] Zhu K.Y., Palli S.R. (2020). Mechanisms, Applications, and Challenges of Insect RNA Interference. Annu. Rev. Entomol..

[63] Wang A., Wang Y., Wang C., Cui B., Sun C., Zhao X., Zeng Z., Yao J., Liu G., Cui H. (2018). Research progress on nanocapsules formulations of pesticides. J. Agric. Sci. Technol..

[64] Yan S., Jiang Q., Shen J. (2022). Research actuality on synergistic mechanism of nanopesticides and their carriers. J. Plant Prot..

